# Protecting the psychological wellbeing of staff exposed to disaster or emergency at work: a qualitative study

**DOI:** 10.1186/s40359-019-0360-6

**Published:** 2019-12-10

**Authors:** Samantha K. Brooks, Rebecca Dunn, Richard Amlôt, G. James Rubin, Neil Greenberg

**Affiliations:** 1 Department of Psychological Medicine, King’s College London, Cutcombe Road, London, SE5 9RJ UK; 2 Public Health England, Emergency Response Department Science & Technology, Health Protection Directorate, Porton Down, Salisbury, Wilts SP4 0JG UK

**Keywords:** Disasters, Employees, Mental health, Qualitative research, Psychological impact

## Abstract

**Background:**

Disasters are becoming more prevalent across the world and people are frequently exposed to them as part of their occupational groups. It is important for organisations to understand how best to support employees who have experienced a trauma such as a disaster. The purpose of this study was to explore employees’ perceptions of workplace support and help-seeking in the context of a disaster.

**Methods:**

Forty employees in England took part in semi-structured interviews. Thematic analysis was used to extract recurring themes from the data.

**Results:**

Participants reported both positive and negative psychological outcomes of experiencing a disaster or emergency at work. Most had little training in how to prepare for, and cope with, the psychological impact. They perceived stigma around mental health and treatment for psychological issues which often made them reluctant to seek help. Many reported that the psychological support available in the workplace was insufficient and tended to be reactive rather than proactive. Interpersonal relationships at work were viewed as being important sources of support, particularly support from managers. Participants suggested that psychosocial training in the workplace could be beneficial in providing education about mental health, encouraging supportive workplace relationships, and developing listening skills and empathy.

**Conclusions:**

Organisations can take steps to reduce the psychological impact of disasters on employees. This could be done through provision of training workshops incorporating mental health education to reduce stigma, and team-building exercises to encourage supportive workplace relationships.

## Background

Trauma-exposed populations are frequently a topic of scholarly discussion, particularly in recent years following the rise of transnational terrorism [1]. With much research focused at an individual level [2], less attention has been paid to the group level, neglecting that many individuals experience trauma together. Commonly people are exposed to disasters as part of an occupational group: for example, emergency services personnel and rescue workers, but also groups such as healthcare workers who assist with emergency response and commercial organisations affected by terrorist attacks or natural disasters. With catastrophic events becoming more prevalent worldwide, it has been suggested that all organisations should ensure they are prepared for disasters as they may impact on staff wellbeing [3]. Understanding the ability of people in a group to effectively respond to such threats is imperative, as the safety and wellbeing of those affected is dependent upon it [4].

Disasters can impair the functioning of affected organisations [5]. Some organisations, especially those with emergency workers and other healthcare professionals, require their staff to continue to function and carry out their role, managing increasing need for their services and for information, whilst dealing with their own personal situations and emotions. It is important for organisations to create a healthy work ethos and environment during crises and also to have systems in place to deal with subsequent distress and disorder.

Literature on the mental health of people in regularly trauma-exposed roles suggests that such individuals are at considerable risk of psychological problems: high rates of post-traumatic stress disorder (PTSD), depression, anxiety and other mental health problems have been observed in rescue workers [6], police [7], body handlers [8] and fire-fighters [9]. The prevalence of post-traumatic stress in these groups varies widely however [10] and scientific reviews suggest that the psychological impact of traumatic exposure can depend on factors such as extent of exposure, social support, and training [11, 12].

Employees not routinely exposed to trauma can also be psychologically affected if they experience a disaster: for instance high rates of distress and mental health problems have been noted in factory workers who experienced an earthquake [13], bank employees who experienced a robbery [14] and Pentagon employees who were working at the time of the September 11th terrorist attacks in New York [15]. A systematic review has shown that factors affecting the extent of the psychological impact in such employees are similar to those affecting professional rescue workers [16]. Research suggests that good organisational leadership and a supportive work culture in general [5] and substantial disaster preparation and planning [17] can have a positive impact upon the wellbeing of staff members prior, during and subsequent to an incident.

However, it is not always clear how best to support trauma-exposed employees: for example, there has been much contention about the effect of ‘debriefing’ – gathering together affected employees following a disaster to discuss the experience – with suggestions that psychological debriefing can be unhelpful or even harmful [18]. Consequently, National Institute for Health and Care Excellence guidelines [19] recommend that such debriefings should not be used. Limited research on psychological interventions for trauma-exposed staff has been carried out and findings are inconsistent [20]. The lack of empirical research on how to best manage trauma-exposed employees means that organisations looking for guidelines on how to support their staff after a disaster are likely to find little evidence of effective interventions.

This study aimed to provide an understanding of how best to support employees after a disaster, in order to inform the development of future psychological interventions for trauma-exposed organisations. To identify what would be needed from a workplace intervention, this study interviewed employees to explore their perceptions of how they may be psychologically affected by a disaster or emergency at work; explore their views on support offered by their workplace; identify factors affecting the likelihood of traumatised employees seeking help; and understand what they would find beneficial in terms of post-disaster workplace interventions.

## Methods

### Design

The study used semi-structured qualitative interviews.

### Participants

Eligible participants had to be aged 18 or over, and currently employed in the United Kingdom (UK). The study aimed to recruit at least ten employees from each of the following sectors: healthcare, emergency services, and commercial organisations in order to ensure the inclusion of insights from a wide variety of employees. We aimed to include those regularly trauma-exposed and those unlikely to have experienced a major incident at work to ensure our results were widely applicable to UK workplaces.

### Procedure

The researchers sent study information letters to the Police Federation, two police constabularies in the south of England, and two doctors’ surgeries identified through personal contacts. The study was advertised in an email circular which reaches all staff and students of our university, the Business Continuity Institute’s newsletter, and on the Gumtree.com website. The authors also used a modified form of snowball sampling, where personal and professional contacts helped find potential participants by recommending additional organisations or individuals. This allowed us to recruit participants we would not have had access to through other methods. Those who were interested in taking part after reading the information sheets contacted the researchers directly.

### Interviews

An interview guide was developed by the researchers, with central questions to be asked in each interview relating to perception of risks in the workplace, disaster preparedness, and experience of traumatic incidents at work. Participants were aware that ‘disasters or emergencies’ were the focus of the research, but in terms of talking about their own experiences, those without involvement in a major incident were encouraged to both consider how they might be affected in a hypothetical disaster/emergency and to discuss any incidents in their workplace which a) were perceived as traumatic or distressing and b) affected more than just the individual. The interviewer informed participants that the results of this study would aid in the development of a psychosocial training package designed to enhance psychological resilience in the workplace. Interviews were carried out by two researchers - SKB (*n* = 31) or RD (*n* = 9), between April 2015–May 2016. Of the 40 interviews, 36 were telephone interviews while 4 were carried out face-to-face. Interviews lasted an average of 60 min (median: 53). They were audio-recorded and transcribed verbatim. Transcripts were stored and coded on NVivo software (QSR International Pty Ltd., 2012) [21].

### Ethics

All participants received information sheets and signed an informed consent form prior to participating. The research was approved by the Psychiatry, Nursing and Midwifery Research Ethics Subcommittee at King’s College London (ref PNM/14/15–29).

### Analysis

Data were analysed inductively according to the principles of thematic analysis [22] using the six-stage approach recommended by Braun and Clark [22]. After multiple readings of the transcripts to allow familiarisation with the data (Stage 1: Familiarisation), transcripts were imported into NVivo where they were broken down into ‘chunks’ of data based on content and labelled with codes, initially by SKB, and then discussed with other members of the team (NG, GJR) (Stage 2: Generating initial codes). Next, codes were collated into potential overarching ‘themes’ and data reflecting the same themes were grouped together, again initially by SKB and later discussed with NG and GJR (Stage 3: Searching for themes). A deeper review of the themes was then carried out, ensuring they reflected the dataset (Stage 4: Reviewing themes) and the themes were then named and given clear working definitions to capture their content (Stage 5: Defining and naming themes). Finally, quotes illustrating each theme were selected for inclusion in this manuscript (Stage 6: Producing the report).

Both interviewers were well-acquainted with the general literature related to traumatic stress and employment – however, as experienced qualitative researchers, used open non-leading questions to gather data, and analysis was based solely on the gathered transcripts rather than utilising any information on the topic which the researchers were aware of before carrying out the interviews. At all stages, the authors discussed the data and the themes to ensure the analysis presented in the current paper reflected the dataset appropriately. Reflexivity was important throughout, with the researcher continuously reviewing the research process and reflecting on how their own experiences may have influenced their interactions with participants or interpretation of the data.

## Results

Participant information is presented in Table 1.

**Table 1:** Participant characteristics

Participant group	Mean age, years (range)	Gender	Mean years in current role (range)	N(%) with experience of traumatic incident	Occupational role or field
Commercial sector	42.0 (21–62)	60% male, 40% female	6.41 (7 months-26 years)	53.3%	Education (*n* = 3), media (*n* = 2), admin (*n* = 2), finance (*n* = 2), legal (*n* = 1), victim support (*n* = 1), fitness (*n* = 1), customer service (*n* = 1), engineering (*n* = 1), business continuity (*n* = 1)
Healthcare	45.3 (24–63)	20% male, 80% female	8.23 (1.5–29)	33.3%	General practitioner (GP), nurse or consultant: *n* = 7 Administrative staff at GP surgery: *n* = 8
Emergency services	44.3 (33–50)	80% male, 20% female	16.35 (2–25)	100%	Police: *n* = 6 Ambulance: *n* = 2 Fire: *n* = 2

Four main themes were identified: the psychological impact of disasters/emergencies; stigma around mental health and help-seeking; support in the workplace (with sub-themes of pre-disaster training, post-disaster support, and workplace relationships); and suggestions for how workplace support could be improved (sub-themes: reducing stigma and psychosocial training package).

Each theme is discussed and illustrated by quotes from the interviews. Participants have been given unique identification numbers. ‘C’ indicates a commercial sector employee, ‘H’ indicates a healthcare professional and ‘E’ a member of the emergency services.

### Psychological impact

Several participants reported positive consequences of experiencing such an incident: for example, a *‘massive boost in their morale and confidence’* (E9) if they had responded well; a new appreciation for life; and greater emotional maturity, compassion, sympathy and understanding of people in difficult circumstances. Experiencing a disaster could also have a positive impact on a team of colleagues; if they responded well together during the incident, this strengthened bonds between colleagues and led to *‘mutual understanding’* which *‘nobody outside that circle really understands’* (E3).

However, the most frequently reported emotional reactions were negative: shock, helplessness, worries about colleagues, fear of future incidents and guilt. Some suggested they could avoid being overly emotionally affected by detaching themselves from the situation. This approach had particular salience in the accounts of emergency services personnel, but participants in other roles also cited deliberate detachment as a way of not becoming too emotionally affected. Spending time with colleagues and using humour were cited as ways of distancing oneself from the horrors of a traumatic incident.

Participants suggested that the level of emotional impact could differ depending on various factors, such as the severity of the event and the disaster typology, with human-initiated incidents inciting more feelings of anger than natural disasters. Emotional impact was also worsened by repeated exposure to media coverage of the incident; seeing television coverage even years later could *‘bring back a lot of horrible memories’* (C9). Participants were more likely to feel traumatised if they identified in some way with the victims of the incident or could draw parallels between the victims and their own family members. Finally, it was suggested that the cumulative effect of multiple different stressors created psychological problems, rather than a single incident itself; other everyday life stressors added to the emotional distress.

### Mental health stigma

Many participants felt their organisations in general did not have good understanding of psychological issues: *‘there’s a surprising amount of almost suspicion about disclosing anything related to mental health’* (C14). Many perceived a lack of understanding from colleagues – *‘their understanding of people is not so great, their empathy is not so great’* (C9). Participants reported feeling concerned that if they spoke up about feeling traumatised, others would view them as creating problems or as *‘blowing things out of proportion’* (C15). As a result, it was common not to speak out until problems were severe: *‘when I did actually go, it was at the point where I couldn’t function any more’* (C10).

For many, the reluctance to speak out was due to fear of being seen as ‘*weak or pathetic’* (C7) and concern that others at work may look down on them. Such concerns were notable in participants who worked with organisations such as the police force or military, but were not members of these organisations themselves; they perceived their colleagues thought they should *‘man up and get on with it’* (C6); *‘there is this expectation that you’ll be kind of resilient and tough’* (C10) and *‘if you ask for help, you therefore must be weak’* (E10). In most cases, these concerns were rooted in the participants’ own perceptions and expectations of stigma rather than anything which had actually been said to them; however, a minority of participants reported that their colleagues had reacted negatively to them seeking help: *‘I’ll come back [from a mental health appointment] and then people will turn round and go, oh you still sane are you?, and you know there’d be quite a few comments made’* (E10).

There was often ‘bravado’ around wanting to be seen as strong, which could also affect the kind of workplace training received and the way in which employees participate in such training. For example, one participant reported a lack of training for the psychological impact due to the ‘bravado’ of the organisation: *‘it’s always that five minutes at the end of the lesson you really don’t want to talk about because we’re all big rufty tuftys and we can all deal with it, it’s that bravado about it’* (E7).

Participants regularly exposed to traumatic incidents reported being afraid they would no longer be chosen for such jobs if their employers thought they had suffered psychologically: *‘it’s quite possible that the organisation could withdraw you from the role that you’re in rather than support you’* (E2). This often led them to avoid admitting to needing support.

### Workplace support

#### Pre-disaster training

Participants from commercial organisations reported that they received practical training on what to do in case of emergencies, but no training on psychological issues; *‘training is more around the physical aspects of getting the people out of the building. There’s no training around what the mental impact could be’* (C2). Some believed this lack of preparation for psychological distress was due to managers not fully appreciating the psychological impact of disasters, while others felt that their workplace did not have anyone with appropriate expertise to advise on mental health. In some cases, even when training time was dedicated to the psychological aspects of disasters, this was seen as unrealistic and lacking in *‘clear learnings or objectives’* (E4), usually because it did not involve interactive learning.

Many participants felt it would be beneficial to receive psychological training in order to be aware of potential risks, recognise the signs of distress, and feel able to admit to struggling.

#### Post-disaster support

Many participants, from all sectors, suggested organisations were better at providing support post-incident than preparing people beforehand: *‘they don’t kind of talk about that beforehand although there is a kind of ( …*) *process afterwards, ( …*) *saying, you know, are you doing okay’* (C10).

Many participants reported that they would not seek help for trauma-related psychological issues. One reason for failing to use support services was lack of awareness – several suggested that they were not made aware of what support was available and believed that raising awareness would encourage help-seeking. Another reason for not seeking support was lack of time; participants – particularly in the medical field - prioritised work demands over their mental health. Other participants cited day-to-day pressures taking priority over seeking psychological support: *‘I think other pressures make it difficult, workload pressures, time pressures, home pressures. People don’t necessarily prioritise themselves’* (E3).

Several participants who had experienced traumatic events had gone through a review or ‘debriefing’ process following the incident. Such processes were often seen as positive, simply because they allowed employees to see that their organisation acknowledged their experience: *‘it’s just the feeling that your organisation kind of gives a damn about you’* (C7). Other services included counselling, occupational health, employee assistance programmes, or links with outside organisations providing support. Participants who had sought help through counselling at work generally spoke of it positively, suggesting it could help them accept and process their experience. However, though support was good, there was little continuity: *‘The support’s good but I think it peters out quite quickly’* (E3).

For many participants, support following an incident was more likely to focus on physical trauma as it could be observed and treated, but *‘the mental trauma isn’t even picked up until later down the line’* (H1).

#### Workplace relationships

Participants felt it was important they were able to support, and be supported by, their colleagues. Many believed they would be able to recognise problems in colleagues due to noticing changes such as increased irritability, seeming distracted or being more quiet than usual; *‘you can tell when someone’s not their usual self’* (C4). Comparing others’ behaviour to their normal behaviour appeared to be the main way of recognising there may be a problem. However, some felt it was difficult to recognise symptoms of trauma in their colleagues or employees as *‘a lot of people hide their feelings’* (C13). Participants from all sectors commented that being close to others within the team, and knowing what symptoms to look for, would make it easier for problems to be recognised.

It was important for participants to feel their managers were approachable and sympathetic. Managers who took the time to *‘check in with you ( …*) *call you and see how you’re doing’* (C10) were praised, as were those who recognised their employees’ needs in terms of time off or being able to work from home. However, several participants reported feeling unsupported by managers following traumatic incidents - *‘It was like a shrug off, oh well it’s happened, it was that type of attitude’* (E5). Managers were seen as unsupportive when they did not communicate enough with their employees after incidents; for example, by not contacting them while they were off sick, or not acknowledging the experiences they had gone through.

Several participants felt that managers would be supportive if they developed problems, but that problems were not spotted at an early stage. Managers taking a more proactive approach to looking after the wellbeing of their staff was considered as possibly being helpful, while reactive approaches were generally spoken of negatively. Participants would prefer a *‘systematic kind of checking once every few weeks or months to see how you were doing psychologically’* (C7).

In general, a supportive workplace atmosphere and close relationships with colleagues were seen as essential. Participants gave several examples of positive workplace environments, such as knowing *‘there’s always somebody to talk to if you’ve had a bit of a stressful day’* (H1), being able to be honest about feelings, feeling listened to, and a generally relaxed atmosphere. Team bonding days were seen as useful ways of encouraging this kind of atmosphere.

### Suggestions for improvement

#### Reducing stigma

Most participants agreed that it was essential for mental health stigma to be reduced. Some reported this was already starting to happen; *‘I think slowly people are beginning to understand that it is something that needs to be looked at and dealt with’* (C7). The best ways of reducing stigma were believed to be raising awareness of mental health issues and *‘telling people that it’s quite normal to feel that way and have those feelings’* (C10). Several participants had seen seniors in the organisation, or individuals who had been in similar roles to themselves, giving talks at the workplace about their experiences and speaking openly about feeling traumatised and needing support. This was seen as helpful in assuring them that their responses were normal and provided employees with positive role models; it *‘really changed people’s perceptions’* (C7).

#### Psychosocial training package

Participants were asked if they had any suggestions about the delivery or content of a workplace psychosocial intervention. Many suggested they would like training in listening skills and being able to recognise trauma symptoms in others. They felt it was important to be educated about where to signpost others for help, and the intervention should make support pathways clearer. Education about trauma and its effects was seen as important. Participants who were regularly trauma-exposed also felt it was important to be educated about the effects of cumulative stress: *‘Make the point that it could be the smaller jobs that could build up. So the drip-drip effect, as well as the sort of one-off major incidents’* (E4).

Several participants suggested that psychoeducational training could be appropriately incorporated into their existing training. Participants from all sectors reported that they had regular ‘training days’ at work or allotted time dedicated to individual training, in which it might be possible to incorporate psychosocial aspects. Several healthcare workers referred to ‘protected learning time’, in which the surgery was closed for emergency appointments only and employees were given several hours in which to participate in training or learning exercises. Participants from commercial organisations reported having health and safety training days, which psychoeducation *‘could be quite interesting to introduce into’* (C5).

Participants suggested various methods of delivery of such a training package. Several felt that training should be delivered either online *‘because they can do it at their own convenience’* (H12) or via educational leaflets *‘rather than finding the time to spend on a day course’* (C13). However, most believed that to really benefit from such a training package they would need an in-person course, at least initially. Some participants had received online training in the past and found it unhelpful, because *‘you’re doing it on your own, and it’s on a computer, and you’re not really paying a hundred per cent attention’* (C3). It was felt that in-person courses would be more accepted *‘because people would think and feel like it’s part of their training ( …*) *people tend not to do things unless they’re forced to’* (C5). These participants felt that online training might be helpful as a follow-up – *‘to reinforce something, but I wouldn’t suggest it as an initial thing’* (C3).

It was important to many participants that training sessions be interactive and encourage active participation from the employees; *‘to involve them and get them to do the talking’* (E4), such as discussions and role-playing scenarios.

Many suggested that several hours spent on psychosocial training would be more useful than a whole day or two days: *‘little chunks are sometimes better ( …*) *rather than a full-on day’* (H9). Several participants suggested that such training should be ongoing, with refresher training at regular intervals.

## Discussion

This study explored views about the psychological impact of disasters and post-incident workplace support. Of interest is that there were few differences in the responses from emergency services personnel, healthcare workers and commercial organisation employees even though emergency services personnel had, understandably, experienced more emergency training and more traumatic incidents.

Our findings supported previous research suggesting that symptoms of trauma can be worsened by exposure to media coverage of the event [23, 24], poor workplace support [5], identification with victims or survivors [25, 26] and the cumulative effect of being regularly exposed to trauma [6, 27]. Post-traumatic stress symptoms resulting from exposure to repeated ordeals have been referred to as ‘Type II Trauma’ [28] and observed in occupational groups who are regularly exposed to traumatic material over time such as those working with traumatised children [29].

Deliberate detachment was reported to be a way of lessening the emotional impact, which may be a useful defence mechanism but only to an extent; avoidance of thinking about the incident at all can worsen distress [30, 31] while confrontive coping – that is, a coping style involving directly confronting the trauma – tends to be associated with more positive outcomes than avoidant coping [32]. Research on rescue workers suggests that deliberate distancing from a traumatic event may be adaptive in the immediate aftermath but is detrimental to recovery if prolonged [33].

Despite the negative impacts described, participants also reported potential positive impacts of being involved in traumatic incidents. This supports previous literature on post-traumatic growth, which has shown that disasters can lead to greater appreciation of life [34] and greater confidence and self-esteem [35].

Many participants were concerned they would be seen as weak, reporting feelings of shame and embarrassment about admitting to psychological problems, and reporting concerns about impact on their career due to lack of understanding by managers or colleagues. Similar feelings of shame about suffering from psychological problems and concerns about impact on career have been noted in doctors [36] and the military [37, 38]. As a result of these barriers, participants often waited until problems were severe before seeking help. Similar findings have emerged from qualitative research on doctors with mental health problems, who tended to delay help-seeking until problems were too severe to ignore [39]. It may be that perceptions of stigma from others could be internalised negative self-perceptions, or ‘self-stigma’ [36] and so interventions aimed at addressing stigmatising beliefs should incorporate this. A review of stigma and barriers to care in military populations [40] suggested that failing to seek help for psychological problems came from three main areas: internal stigma (negative perceptions of oneself as a result of experiencing mental health problems), external stigma (negative perceptions from others) and access factors such as not knowing what services are available. Our results certainly supported the idea that difficulties in accessing professional support and stigma are the main barriers to help-seeking, although it was difficult to assess the extent to which external stigma was problematic. A minority of participants did report experiencing negative reactions from others, but many simply reported that they expected others would see them as weak, which may be a result of self-stigma. We suggest that the issue of stigma appears to be somewhat circular, in that employees felt ashamed to talk about their concerns as they feared being judged, but a lack of openness is likely to perpetuate stigma and lead individuals to hold stigmatising views. It may be useful for further research to address the distinction between internal and external stigma and explicate their relationship with help-seeking in trauma-exposed organisations.

Of interest is that even though some participants had received emergency-focused training, this tended to neglect the psychological aspects of dealing with traumatic events; when a psychological element was incorporated, this was often viewed as unrealistic or not aimed at the right level, suggesting there is currently a major gap in the training employees receive. This is perhaps unsurprising as a recent report [41], surveying over 400 employees from a variety of organisations, found that more than half reported no mental health and wellbeing training was available for managerial staff. A review of workplace psychosocial training and interventions specifically in the context of a disaster [20] revealed a striking lack of evaluations of such programmes; overall it appears there is an urgent need for more research to ascertain the best ways of providing organisational training with a psychological element.

Participants felt it was important for organisations to foster a supportive atmosphere at work and wanted to be able to support and feel supported by their colleagues. It is interesting that several participants felt confident recognising symptoms of distress. Evidence suggests that these can be difficult to detect: for example, studies of primary care show that practitioners find it difficult to recognise symptoms of anxiety and depression in their patients [42, 43]. It is possible that employees may be unhelpfully overestimating their ability to detect distress in colleagues - this is a topic that is worthy of further exploration. Research on military populations [44] and student populations [45] has shown that many people only choose to seek treatment on the advice of friends, colleagues or family members, suggesting that peers can play an important role in help-seeking. Interventions should therefore aim to train employees on how to recognise signs of distress.

Participants suggested that managers should be good listeners, approachable, recognise the needs of their employees and take a proactive approach to checking on the wellbeing of their teams. However, often managers were seen as unsupportive or too busy to be able to stay aware of their employees’ wellbeing. We suggest it may be useful to provide managers with education about mental health and highlight the importance of a proactive approach towards their team’s mental health and their allied ability to perform well at work. For example, presenteeism – continuing to go to work while unwell – can have a great impact on productivity and can be costly to the organisation as a whole [46] so it benefits both the individual and their organisation to improve their wellbeing. Research has highlighted the positive effects of supportive work culture, camaraderie between colleagues and supportive leadership [5, 47] and the negative effects that poor workplace relationships and dissatisfaction with leaders can have on those exposed to trauma at work [48, 49]. Military studies also suggest that good leadership and group cohesion are strongly preventative of mental health deterioration [50] and a review highlighted the importance of team cohesion and positive working relationships and recommended training specifically to foster inter-personal skills [51]. Importantly, the need for supportive relationships and good management was not viewed as specific to disasters or trauma; participants talked about wanting the same kind of support in any stressful situation. We suggest that current initiatives to encourage organisations to invest in having a mentally healthy workplace should include taking account of traumatic incidents too.

Some participants suggested that ‘debriefing’ after traumatic events was helpful, although this tended to refer to informal discussions with colleagues rather than formal psychological debriefing provided by professionals. It should be noted that studies on the effectiveness of psychological debriefing have given inconsistent results, with some showing debriefing is harmful [18]. It also appeared that many participants felt the value of debriefing was the acknowledgement of the experience they had been through. Such methods are not recommended in national treatment guidance documents [19]. Workplace counselling services were also generally spoken of positively. However, many participants felt that the focus was on physical trauma rather than psychological, and that organisations were unable to provide adequate psychological support due to having no one trained to recognise such issues.

Our results highlight the importance of reducing stigma and encouraging open communication. Participants were positive about hearing talks from other individuals in their roles who had experienced traumatic incidents and were not ashamed to discuss their subsequent psychological problems or help-seeking; watching videos of such individuals was also useful. Therefore, incorporating talks or videos from people who have been through traumatic situations into training could be helpful. This strategy is known as ‘contact’ and has been shown to reduce stigma around mental illness [52]. Participants also felt it was important that organisations should always be ready to provide appropriate support, rather than having to hurry to put systems into place after an incident, highlighting the importance of being proactive rather than reactive. Overall, our results support the suggestion [3] that experiencing a disaster can impact on wellbeing and that organisations should prepare for supporting their staff so as to minimise the potential negative impact.

Participants felt that training packages encouraging good communication and empathy for others would be helpful. Such training could provide an understanding of mental health problems and risks; information on how to improve listening skills; education about trauma and its effects; and practical information such as where to signpost others for appropriate help. It was suggested that this be incorporated into existing training during the working day or count towards personal learning time. In-person, interactive training with presentations, roleplaying, discussion, and talks from people who have been through traumatic experiences would be useful. Participants suggested this could be supplemented by follow-up refresher training, either via further in-person courses, online courses or supplementary reading material. A training programme which addresses many of these elements (Trauma Risk Management, or TRiM) [53] has been developed for the military and has been successful in reducing mental health stigma and improving employees’ ability to provide support to each other [54–56] in several organisations regularly exposed to trauma. It may be that elements of TRiM could be incorporated into a training package for employees of other, not regularly trauma-exposed organisations in order to prepare them in case such an incident did occur.

### Limitations

Several limitations exist with this work. Firstly, transcripts were coded independently by one author. Though emerging themes and sub-themes were discussed with other members of the team, we did not double code transcripts which may have helped to minimise potential bias. In future, we would use a more formal process of cross-validation between researchers, with several full transcripts double-coded.

The sample size was relatively small, so participants are not necessarily representative of the general working public. There remains debate about the ideal sample size for qualitative research; some researchers argue that data saturation can be reached after as few as six interviews [57] and that smaller numbers are better as the interviewers can build rapport with their participants [58]. Generally, it appears that 25–30 participants is adequate [59], suggesting that the current study’s population of *n* = 40 is an appropriate number for this type of research. In line with all qualitative studies, this paper does not provide any insight into how commonly such themes would be reported in a quantitative prevalence study.

There may have been selection bias in that those who had particularly strong feelings about the topic may have been more likely to volunteer - so awareness of the psychological impact of trauma may be greater in our sample than in the general population. Importantly, stigma may be greater in the wider population, as those who volunteered to participate are clearly comfortable discussing mental health issues.

Our participants had different levels of disaster experience, ranging from exposure to multiple major traumatic events to no experience at all. This was a deliberate choice, as we were interested in exploring both the preparedness of those in organisations not expecting to be exposed to trauma and the experiences of those who were routinely exposed. This could be seen as a limitation in terms of data synthesis; however, we found a similar lack of preparedness and lack of workplace support across all participants, and importantly, similar support needs. This suggests that psychosocial training incorporated into workplace disaster training could be extrapolated to other stressful situations at work; for example, an intervention aimed at educating employees about how to recognise distress and support others would not only help in a disaster but could also be applied to more ‘everyday’ stressors such as bereavement or relationship breakdowns.

Although we were careful to assure participants of confidentiality and anonymity, it is possible some may have been concerned that they would be able to be identified through their responses. Given the importance of confidentiality highlighted by our results and the concerns participants had about talking to others within their organisations about mental health problems, it is possible this may have led participants to downplay certain issues or avoid topics. There may also have been social desirability bias in the participants’ responses, in that they may have felt uncomfortable telling the interviewer any controversial opinions.

Finally, the use of telephone interviews may have influenced the findings. This was done as we recruited participants from all over the UK, and it would have been impractical to carry out all interviews in person. However, we acknowledge that face-to-face interviews may yield different findings due to social cues influencing the relationship between interviewer and participant [60].

### Strengths

Due to the nature of qualitative research and the potential for bias in interpretations of the data, it is important for the researchers to demonstrate that their research is trustworthy [61]. In line with suggestions for writing up qualitative analysis [22, 61] the current paper provides a detailed description in the Methods section of how the analysis was carried out.

To reduce the risk of bias, the quality of the analysis was checked by sending a draft of the manuscript to three participants and asking them to give feedback on whether they felt the analysis reflected their responses appropriately. All three felt their views had been appropriately reported.

Reflexivity was important throughout. The interviewers made notes in NVivo of their observations and perceptions of each interview, immediately after each interview ended so as to avoid recall bias. The interviewers considered their role in data collection and potential for interpreter bias in the analysis, acknowledging that they were actively involved in the interview process and in drawing interpretations from the data. Although the interviewers had experience of disaster research and may have had their own assumptions prior to doing this study, throughout the interviews they consciously questioned their own assumptions and encouraged participants to talk freely about their own experiences and opinions, often following up statements with probing questions to ensure they had understood the responses. The analysis of the data was discussed with other members of the team, who had no part in the data collection and were thus approaching the data with no preconceptions about what the findings might be.

## Conclusions

Despite participants’ acknowledgement that the psychological impact of experiencing a disaster at work could be considerable, few reported any degree of psychological preparedness. Participants were frequently reluctant to seek help from employers in respect of any psychological trauma due to a combination of factors including lack of awareness of support available, not prioritising one’s own mental health, concerns about confidentiality, and a belief that admitting to mental health problems may lead to being seen as weak and potentially impacting their career. Our findings suggest that education about psychological trauma may lead to better understanding, better recognition of symptoms in oneself and in others, less judgement, and therefore reduced stigma, and that positive relationships with others in the workplace can have a positive impact on psychological wellbeing. This review suggests there are several steps organisations could take to benefit their employees’ mental health, and that their disaster planning should include reduction of stigma through education; encouraging employees not to neglect mental health; encouraging open communication about psychological issues at work; improving supportive relationships between co-workers; and educating employees about when and where to seek help.

## Data Availability

The datasets generated during and/or analysed during the current study are not publicly available due to content that potentially identifies participants, but are available from the corresponding author on reasonable request.

## Abbreviations

GP: General practitioner (referred to in Table only)
PTSD: Post-traumatic stress disorder
TRiM: Trauma Risk Management
UK: United Kingdom
